# Development and external validation of a nomogram for predicting preterm birth at < 32 weeks in twin pregnancy

**DOI:** 10.1038/s41598-021-91973-y

**Published:** 2021-06-14

**Authors:** Jun Zhang, Wenqiang Zhan, Yanling Lin, Danlin Yang, Li Li, Xiaoying Xue, Zhi Lin, Mian Pan

**Affiliations:** 1 Department of Obstetrics and Gynaecology, Fujian Maternity and Child Health Hospital, Affiliated Hospital of Fujian Medical University, Fuzhou, 350000 Fujian China; 2 Department of Medical Ultrasonics, Fujian Maternity and Child Health Hospital, Affiliated Hospital of Fujian Medical University, Fuzhou, 350000 Fujian China; 3 School of Public Health, Shanghai Jiao Tong University School of Medicine, Shanghai, 200025 PR China; 4 Department of Obstetrics and Gynaecology, Shengli Clinical Medicine College of Fujian Medical University, Fuzhou, 350000 Fujian China; 5 Department of Obstetrics and Gynaecology, Fujian Maternity and Child Health Hospital, Affiliated Hospital of Fujian Medical University, No.18 Daoshan Road, Fujian, 350001 China

**Keywords:** Diagnosis, Risk factors, Signs and symptoms

## Abstract

The purpose of this study was to develop a dynamic model to predict the risk of spontaneous preterm birth at < 32 weeks in twin pregnancy. A retrospective clinical study of consecutively asymptomatic women with twin pregnancies from January 2017 to December 2019 in two tertiary medical centres was performed. Data from one centre were used to construct the model, and data from the other were used to evaluate the model. Data on maternal demographic characteristics, transvaginal cervical length and funnelling during 20–24 weeks were extracted. The prediction model was constructed with independent variables determined by multivariate logistic regression analyses. After applying specified exclusion criteria, an algorithm with maternal and biophysical factors was developed based on 88 twin pregnancies with a preterm birth < 32 weeks and 639 twin pregnancies with a delivery ≥ 32 weeks. It was then evaluated among 34 pregnancies with a preterm birth < 32 weeks and 252 pregnancies with a delivery ≥ 32 weeks in a second tertiary centre without specific training. The model reached a sensitivity of 80.00%, specificity of 88.17%, positive predictive value of 50.33% and negative predictive value of 96.71%; ROC characteristics proved that the model was superior to any single parameter with an AUC of 0.848 (all P < 0.005). We developed and validated a dynamic nomogram model to predict the individual probability of early preterm birth to better represent the complex aetiology of twin pregnancies and hopefully improve the prediction and indication of interventions.

## Introduction

Complications of preterm birth (PTB) are the primary cause of death among children in the first 5 years of life, accounting for approximately 35% of deaths among newborns and 18% of all paediatric deaths^1^. Twin gestations have increased continuously over the past decades and currently account for 3% of all live births and approximately 15–20% of all PTBs, attributable in large part to the increased use of assisted reproductive technologies^2,3^. Compared with singletons, the neonatal mortality rate is more than fourfold higher in twins. Additionally, the risk of neurologic morbidity in very preterm infants was 4.1% for singleton infants and 15.4% for monochorionic twins^4–6^.

To date, strategies for the prevention of PTB in twin pregnancy, such as the use of vaginal progesterone, cervical pessary and cervical cerclage, remain controversial or are considered to have limited effects^7–15^. This is partly due to results from RCTs using inefficient models of risk assessment that lead to negative results for cervical cerclage, vaginal progesterone or cervical pessary. To address the growing desire for better guidance for clinical practice, it is necessary to distinguish asymptomatic patients who are at greater risk of early PTB from the whole twin-pregnancy population.

There are discrepant opinions on how precisely the risk of spontaneous preterm birth (SPTB) in twin pregnancies can be determined. More importantly, preterm birth is a complex syndrome with many causes and phenotypes. In twins, there is an additional pre-existing risk due to overdistension and the effect on the cervix but possibly also due to increased uterine irritation and subclinical inflammation after ART or physical and psychological maternal stress factors^16,17^. The great variety in PTB rates signifies that there are epigenetic transgenerational stress factors and determinants from the social environment and the health care system^18–20^. In addition, perinatal morbidity and mortality among twins vary by chorionicity, and monochorionicity is significantly associated with an increased risk of PTB^21^.

Previous studies have demonstrated the association between SPTB in twin pregnancies and specific clinical indicators, such as ethnic origin, age, nulliparity, chorionicity, body mass index (BMI), tobacco usage, history of previous preterm delivery, cervical length and funnelling^21–31^. Different combinations of clinical variables might indicate different likelihoods of SPTB. The purpose of this study is to synthesize an array of maternal demographic factors and clinical variables and develop a practical algorithm to calculate the risk of SPTB for twin pregnancies, similar to the first trimester genetic disease screening tools or the Framingham heart disease score^32^.

## Results

### Characteristics of the development and external validation groups

**Figure 1:**
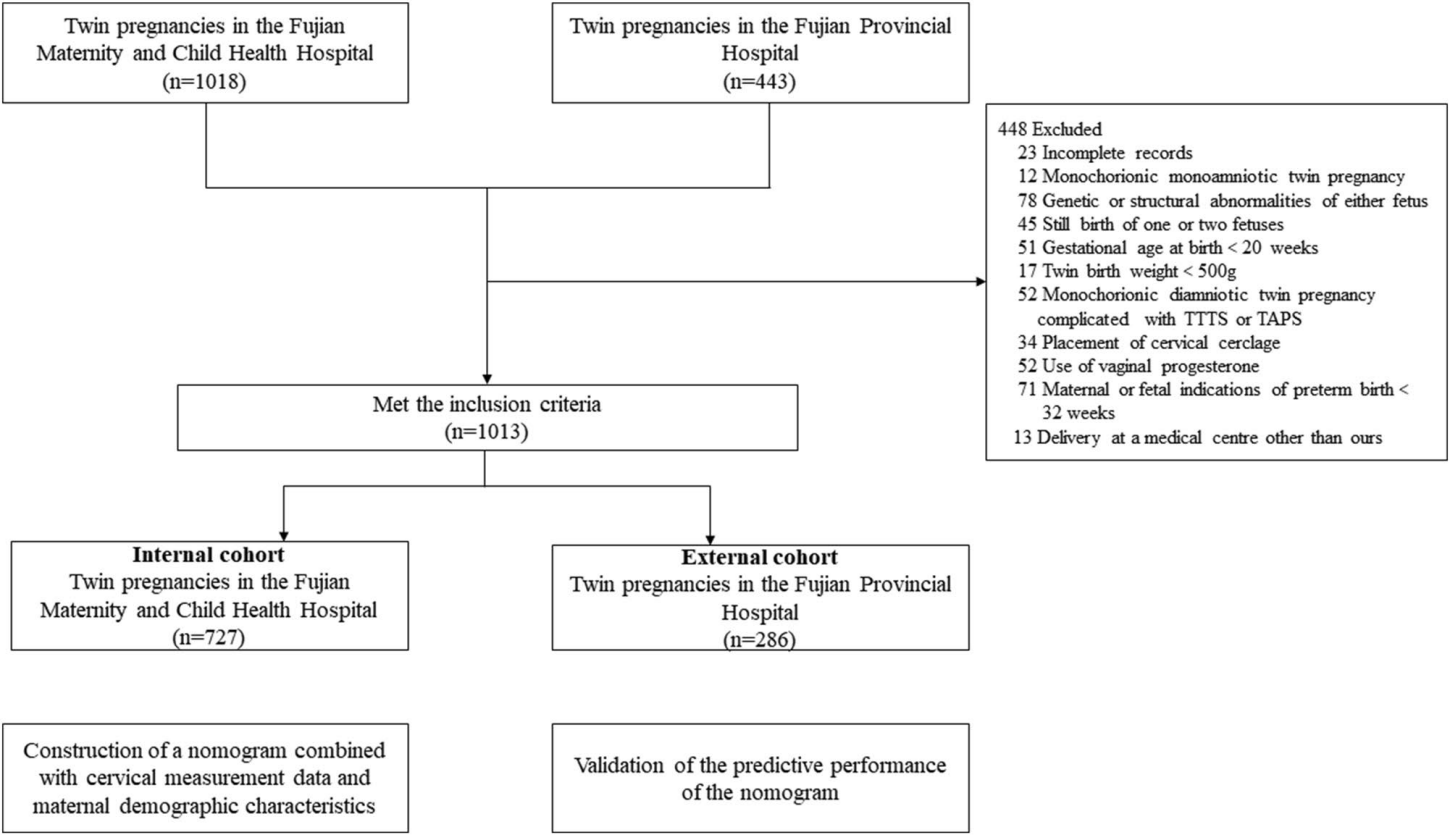
Selection process of subjects.

In total, 1013 asymptomatic twin pregnancies were eligible for the study, of which 727 collected from the Fujian Maternity and Child Health Hospital were assigned to the training group, while 286 from the Fujian Provincial Hospital were assigned to the external validation group (Fig. 1). In the whole study population, the numbers of positive cases of SPTB at < 28, 32, 34 and 37 weeks were 31 (3.06%), 122 (12.04%), 207 (20.43%) and 596 (58.84%), respectively.

There were no significant differences in maternal demographic and clinical characteristics between the training and validation groups (all P > 0.05), indicating that the features of the training and external validation groups were similar and that subsequent external validation would be representative (Table 1).

**Table 1:** Characteristics of Twin pregnant women in training and validation group.

	Training group n = 727	External validation group n = 286	Test statistics	P-value
Age (years)	30.0 (26.0–33.0)	30.0 (27.0–33.0)	− 0.502	0.601
Prepregnancy BMI (kg/m^2^)	24.8 (23.0–25.6)	24.2 (23.2–25.8)	− 0.463	0.592
**Nulliparity**			3.152	0.085
No	408 (56.12)	146 (51.05)		
Yes	319 (43.88)	140 (48.95)		
**Chorionicity**			1.738	0.296
Dichorionic	409 (56.26)	149 (52.10)		
Monochorionic	318 (43.74)	137 (47.90)		
Cervical length (20–24 weeks) (mm)	33.5 (28.0–36.5)	33.0 (27.5–36.0)	− 1.839	0.082
**History of previous cervical surgery**			0.359	0.603
No	710 (97.66)	278 (97.20)		
Yes	17 (2.34)	8 (2.80)		
**Previous preterm birth or late abortion**			3.726	0.061
No	614 (84.46)	254 (88.81)		
Yes	113 (15.54)	32 (11.19)		
**Cervical funneling**			1.321	0.291
No	560 (77.03)	229 (80.07)		
Yes	167 (22.97)	57 (19.93)		
**Smoking**			0.019	0.901
No	716 (98.49)	281 (98.25)		
Yes	11 (1.51)	5 (1.75)		
**Assisted reproductive technology**			0.126	0.709
No	408 (56.12)	163 (56.99)		
Yes	319 (43.88)	123 (43.01)		
**Gestational diabetes**			1.389	0.286
No	555 (76.34)	229 (80.07)		
Yes	172 (23.66)	57 (19.93)		

Data are shown as number (%) or median (IQR).

*BMI* body mass index.

### Predictive factors associated with SPTB at < 32 weeks

**Figure 2:**
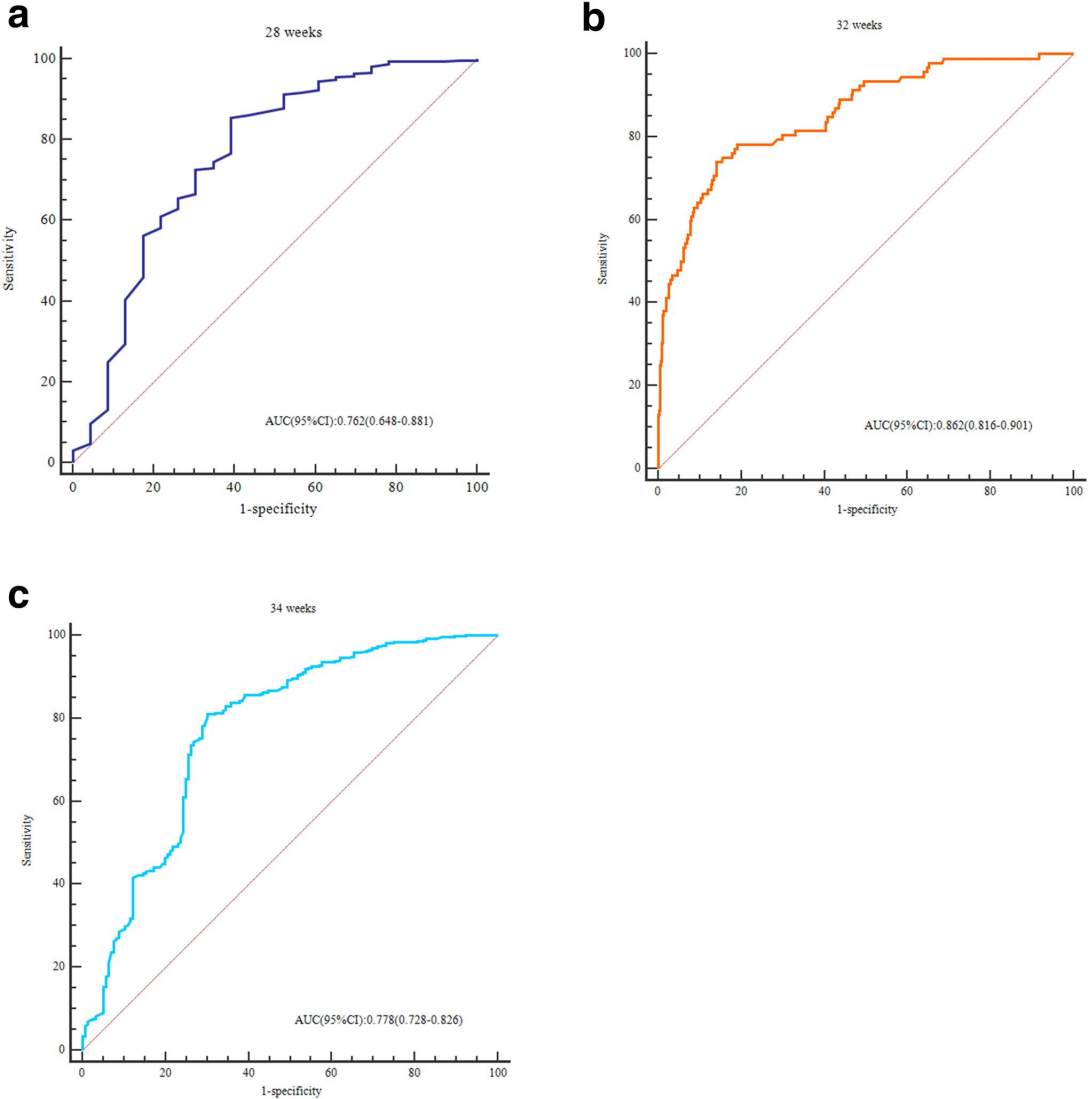
ROC curves for three gestational weeks at delivery (before 28 weeks, 32 weeks and 34 weeks).

In the training group, we conducted univariate and multivariate regression analyses to detect the correlations between clinical variables and probabilities of preterm delivery before 28 weeks, 32 weeks, and 34 weeks by applying the AIC-based backward procedure (Table 2). Then, we constructed three ROC curves for predicting SPTB according to the results of multivariate analysis. By comparing the AUCs, we found that the predictive value for SPTB at < 32 weeks was the highest (Fig. 2). After comprehensively considering the predictive power and the number of positive cases of SPTB before the three gestational weeks, we chose to establish a predictive model for predicting PTB at < 32 weeks. Multivariate logistic regression analysis (< 32 weeks) showed that nulliparity, monochorionicity, lower prepregnancy BMI, previous preterm birth or late abortion, cervical funnelling and shorter cervical length were independent risk factors for SPTB at < 32 weeks.

**Table 2:** Multivariate (adjusted) OR, 95% CI and P values according to the probability of PTB before 28 weeks, 32 weeks, and 34 weeks in the training group (n = 727).

Variable	28 weeks			32 weeks			34 weeks		
	OR (95%CI)	aOR (95%CI)	P value	OR (95%CI)	aOR (95%CI)	P value	OR (95%CI)	aOR (95%CI)	P value
Age (years)	1.02 (0.93–1.12)	1.06 (0.92–1.19)	0.501	0.98 (0.96–1.05)	1.05 (0.96–1.15)	0.282	0.98 (0.95–1.03)	1.01 (0.95–1.06)	0.872
Prepregnancy BMI (kg/m2)	0.69 (0.61–0.82)	0.82 (0.71–1.15)	0.382	0.65 (0.56–0.72)	0.64 (0.51–0.71)	< 0.001	0.78 (0.67–0.86)	0.86 (0.77–0.92)	0.003
**Nulliparity**			0.396			0.001			0.019
No	1.00	1.00		1.00	1.00		1.00	1.00	
Yes	2.45 (1.06–5.89)	1.51 (0.63–4.07)		2.82 (1.80–4.51)	2.83 (1.43–5.46)		2.19 (1.53–3.16)	1.78 (1.17–2.69)	
**Chorionicity**			0.257			0.003			0.398
Dichorionic	1.00	1.00		1.00	1.00		1.00	1.00	
Monochorionic	2.35 (1.02–5.72)	1.79 (0.71–4.92)		3.02 (1.90–4.80)	2.83 (1.45–5.50)		1.63 (1.15–2.28)	1.28 (0.79–1.89)	
Cervical length (20–24 weeks) (mm)	0.83 (0.77–0.89)	0.86 (0.78–0.93)	< 0.001	0.78 (0.72–0.81)	0.82 (0.78–0.89)	< 0.001	0.86 (0.81–0.93)	0.91 (0.86–0.95)	< 0.001
**History of previous cervical surgery**			0.402			0.459			0.313
No	1.00	1.00		1.00	1.00		1.00	1.00	
Yes	1.92 (0.29–15.19)	3.22 (0.29–40.16)		1.52 (0.46–5.26)	2.09 (0.38–12.18)		1.98 (0.75–5.26)	1.89 (0.61–6.52)	
**Previous preterm birth or late abortion**			0.003			< 0.001			0.012
No	1.00	1.00		1.00	1.00		1.00	1.00	
Yes	4.58(1.98–10.71)	4.65(1.72–12.68)		2.91(1.79–4.78)	5.36(2.36–12.23)		1.76(1.12–2.72)	2.01(1.15–3.61)	
**Cervical funneling**			0.006			< 0.001			< 0.001
No	1.00	1.00		1.00	1.00		1.00	1.00	
Yes	13.50 (4.93–36.93)	4.93 (1.61–15.12)		12.39 (7.56–20.31)	7.93 (4.16–15.26)		9.38 (6.31–14.09)	6.08 (3.81–9.26)	
**Smoking**			0.068			0.452			0.863
No	1.00	1.00		1.00	1.00		1.00	1.00	
Yes	6.82 (1.51–33.92)	6.49 (0.96–41.06)		3.72 (1.13–12.72)	2.15 (0.39–12.06)		1.92 (0.53–6.62)	0.89 (0.23–3.63)	
**Assisted reproductive technology**			0.189			0.071			0.702
No	1.00	1.00		1.00	1.00		1.00	1.00	
Yes	0.71 (0.32–1.62)	0.52 (0.19–1.42)		0.92 (0.63–1.61)	0.56 (0.28–1.08)		1.09 (0.75–1.52)	0.93 (0.61–1.39)	
**Gestational diabetes**			0.438			0.409			0.286
No	1.00	1.00		1.00	1.00		1.00	1.00	
Yes	0.66 (0.21–2.01)	0.60 (0.15–2.16)		1.12 (0.69–1.91)	1.18 (0.58–2.52)		1.26 (0.83–1.85)	1.38 (0.86–2.29)	

*OR* odds ratio, *CI* confidence interval.

### Development and validation of a dynamic nomogram for SPTB at < 32 weeks

**Figure 3:**
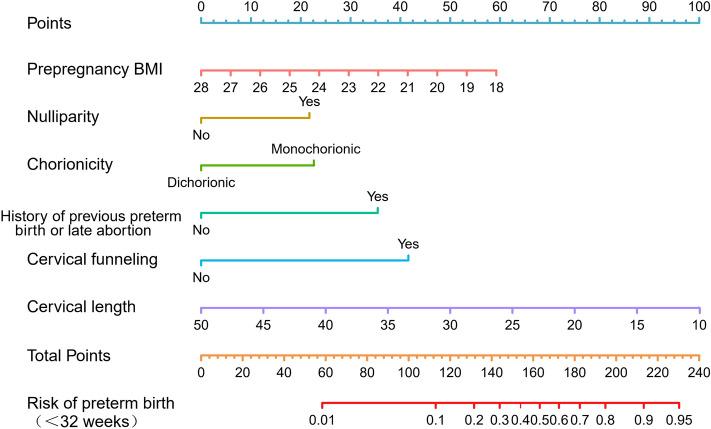
Nomogram for the prediction of PTB < 32 weeks based on six independent risk factors. To calculate the probability of PTB < 32 weeks in twin pregnancies, the point for each variable is assigned by the corresponding value of the "point" axis, and points are plotted on the total points axis. The comprehensive risk of PTB at < 32 weeks for twin pregnancy corresponds to the total points.

Based on meaningful independent factors in multivariate regression analysis, we developed a nomogram to predict SPTB probability at < 32 weeks (Fig. 3). Each point could be determined based on the intersection of the vertical line from the variable to the point axis. Then, the total risk score was calculated by adding each variable point. The possibility of twin SPTB at < 32 weeks could be read on the total point axis.

**Figure 4:**
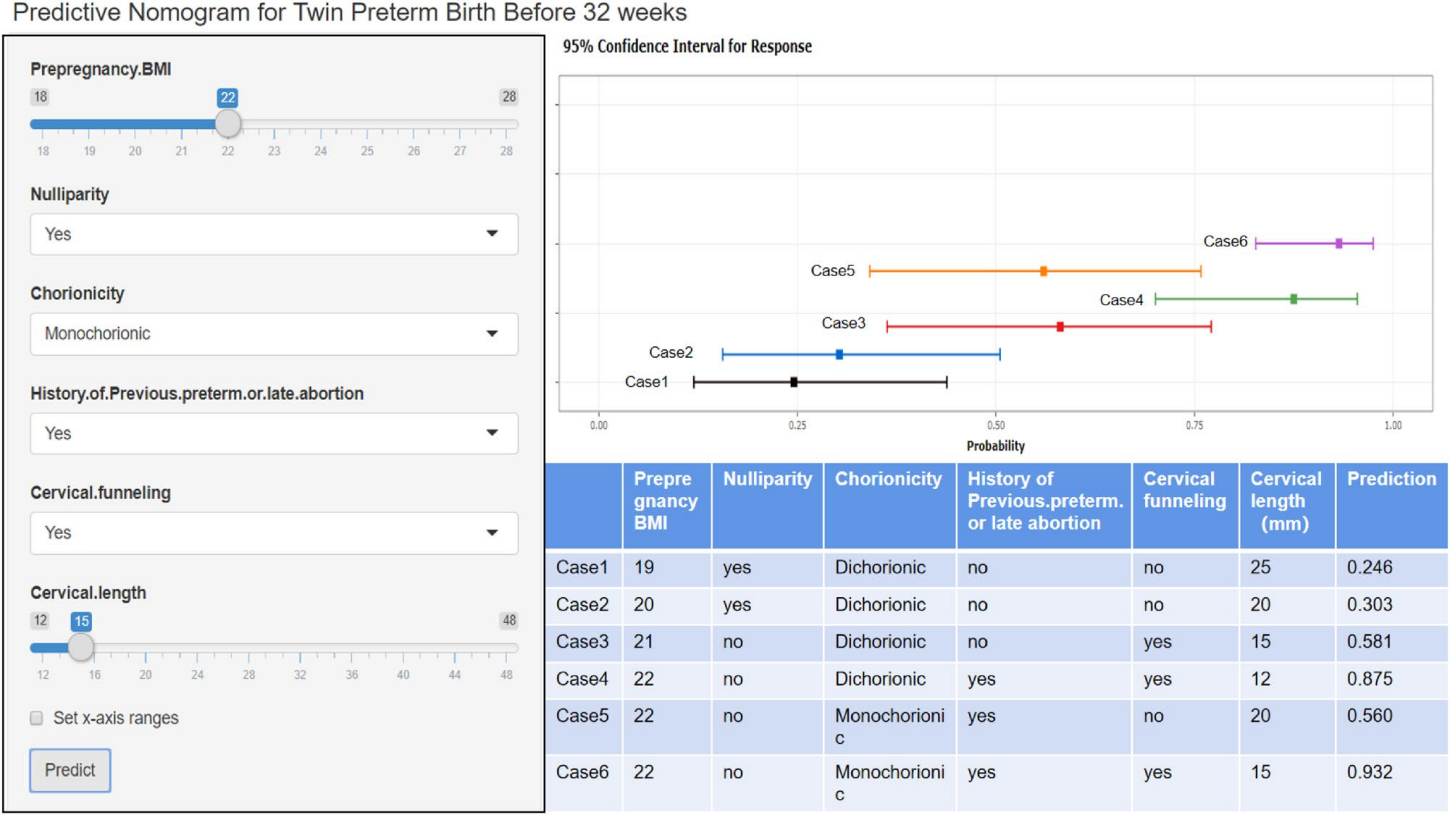
Screenshot of the online user-friendly model (https://zhanwenqiang.shinyapps.io/DynNomapp/) for the prediction of PTB. The users enter variables in the application tool on the left, and then the corresponding predicted probabilities and 95% confidence intervals (CIs) are displayed in the right figure. The table at the bottom right shows six examples of input variables and corresponding predicted probabilities.

**Figure 5:**
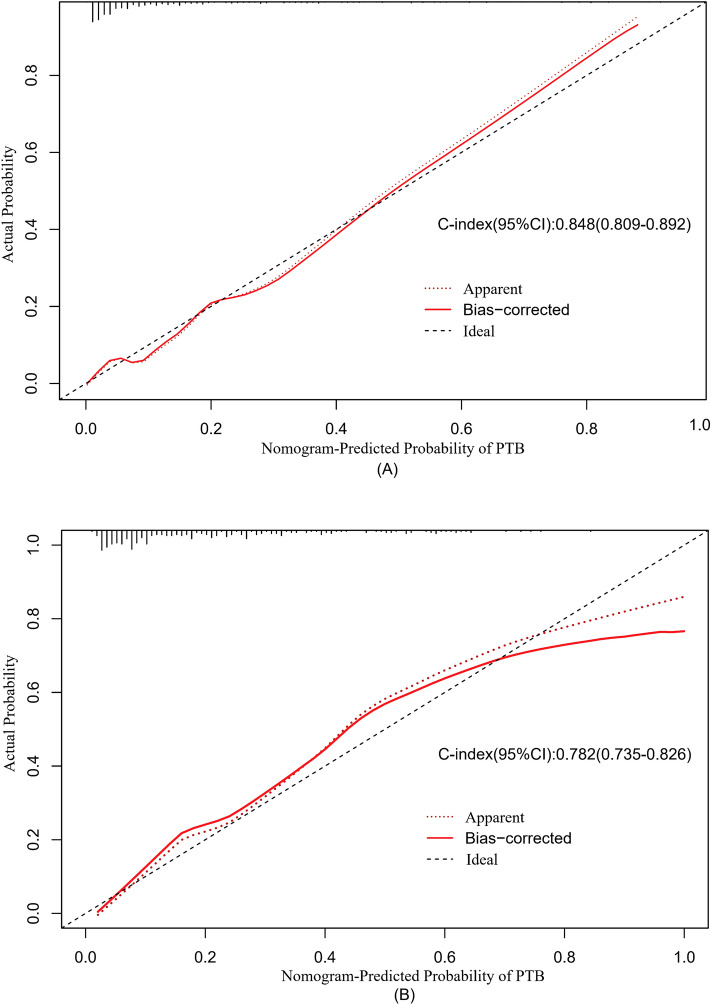
Calibration plots for the predicted and observed overall risk of the nomograms in (**A**) the training group; (**B**) the external validation group. The x-axis demonstrates the nomogram-predicted probability, and the y-axis shows the actual observed probability.

Furthermore, a user-friendly dynamic predicative nomogram was established and is available online (https://zhanwenqiang.shinyapps.io/DynNomapp/). The dynamic nomogram conveniently provided the individual probability of SPTB, which was calculated automatically by the input parameters of each subject (Fig. 4, PS: To facilitate readers’ understanding, we specifically recorded a video of how to use the model, which is in the attachment). Harrell's concordance index value of the nomogram model in the training group was 0.848 (95% CI 0.809–0.892). When applied to the external validation group, Harrell's concordance index value in the external group was 0.782 (95% CI 0.735–0.826). The calibration curves indicated that the probability predicted by the nomogram was in good agreement with the actual probabilities in both the internal cohort and external cohort (Fig. 5).

### Model performance test and risk stratification

**Figure 6:**
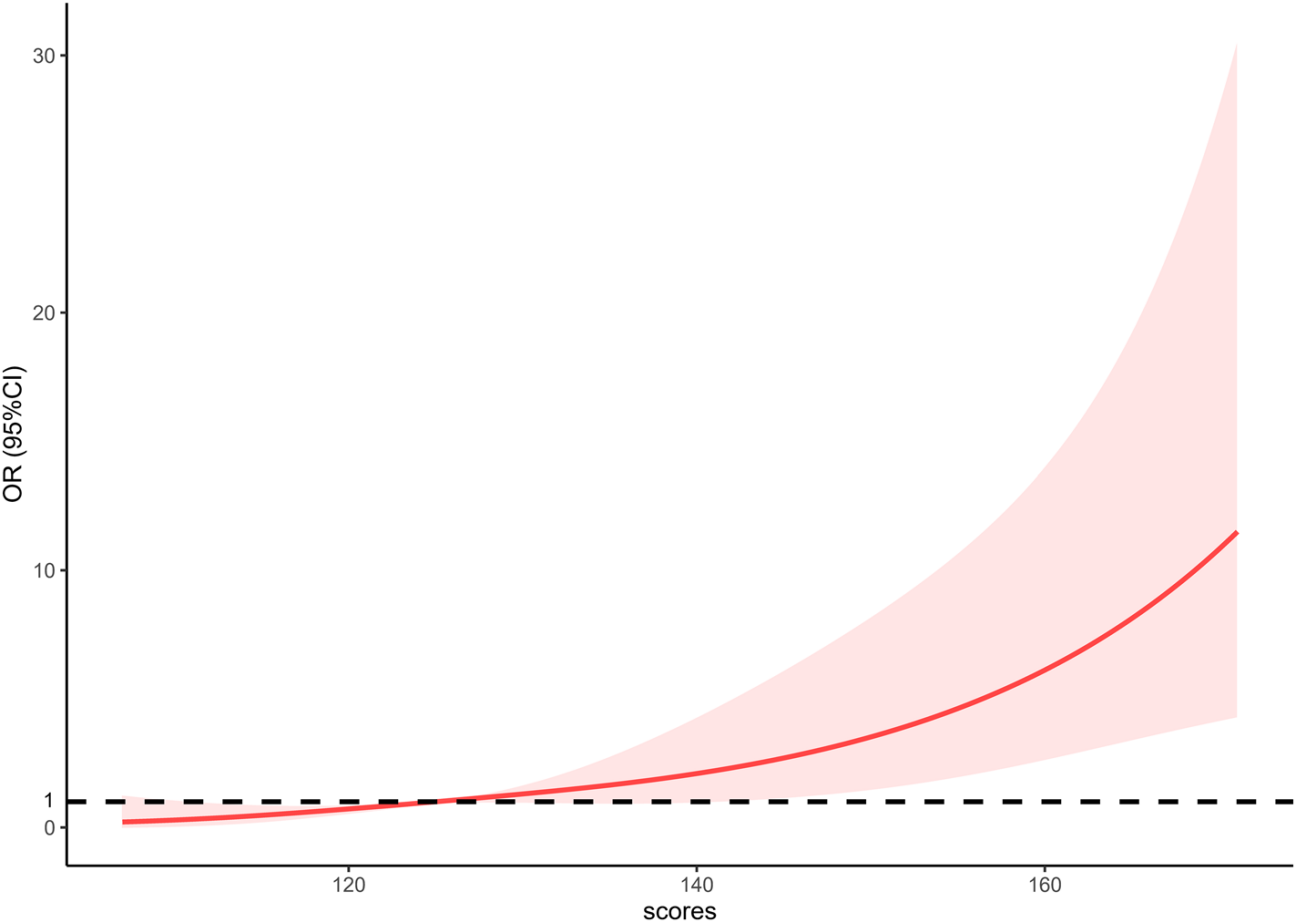
Restricted cubic splines for the nonlinear relationship between the risk of twin preterm birth < 32 weeks and increased risk scores. The solid line displays the odds ratio (OR), and the dashed line represents the 95% confidence interval (CI).

**Figure 7:**
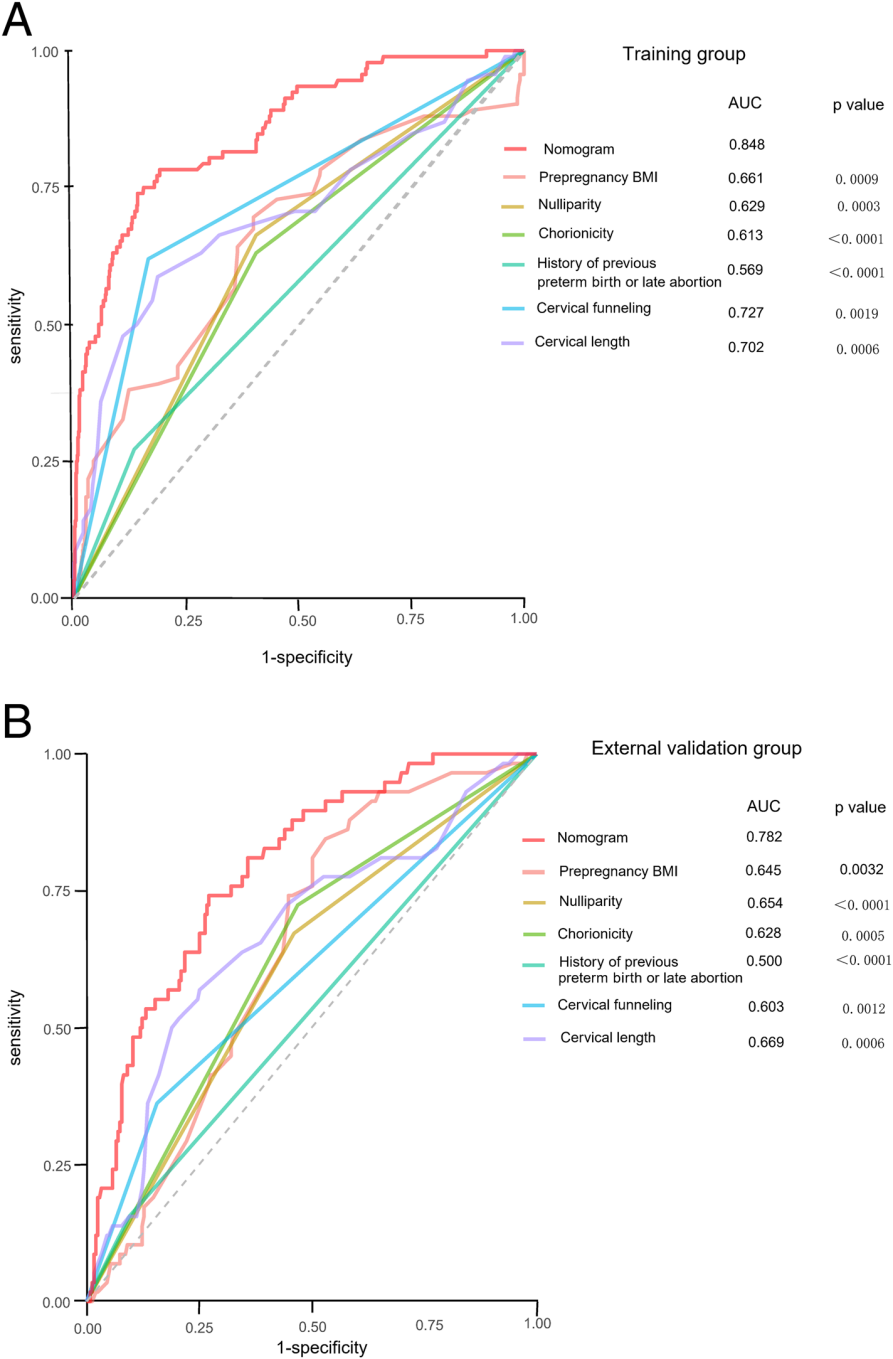
Validation of the predictive accuracy of the nomogram and six individual predictors. ROC curves and AUCs were used to assess the predictive accuracy of the nomogram compared with either meaningful variable (prepregnancy BMI, nulliparity, chorionicity, previous preterm birth or late abortion, cervical funnelling, cervical length). P values show the AUC for the nomogram versus the AUC for other variables alone. *AUC* area under the curve, *ROC* receiver operating characteristic.

**Figure 8:**
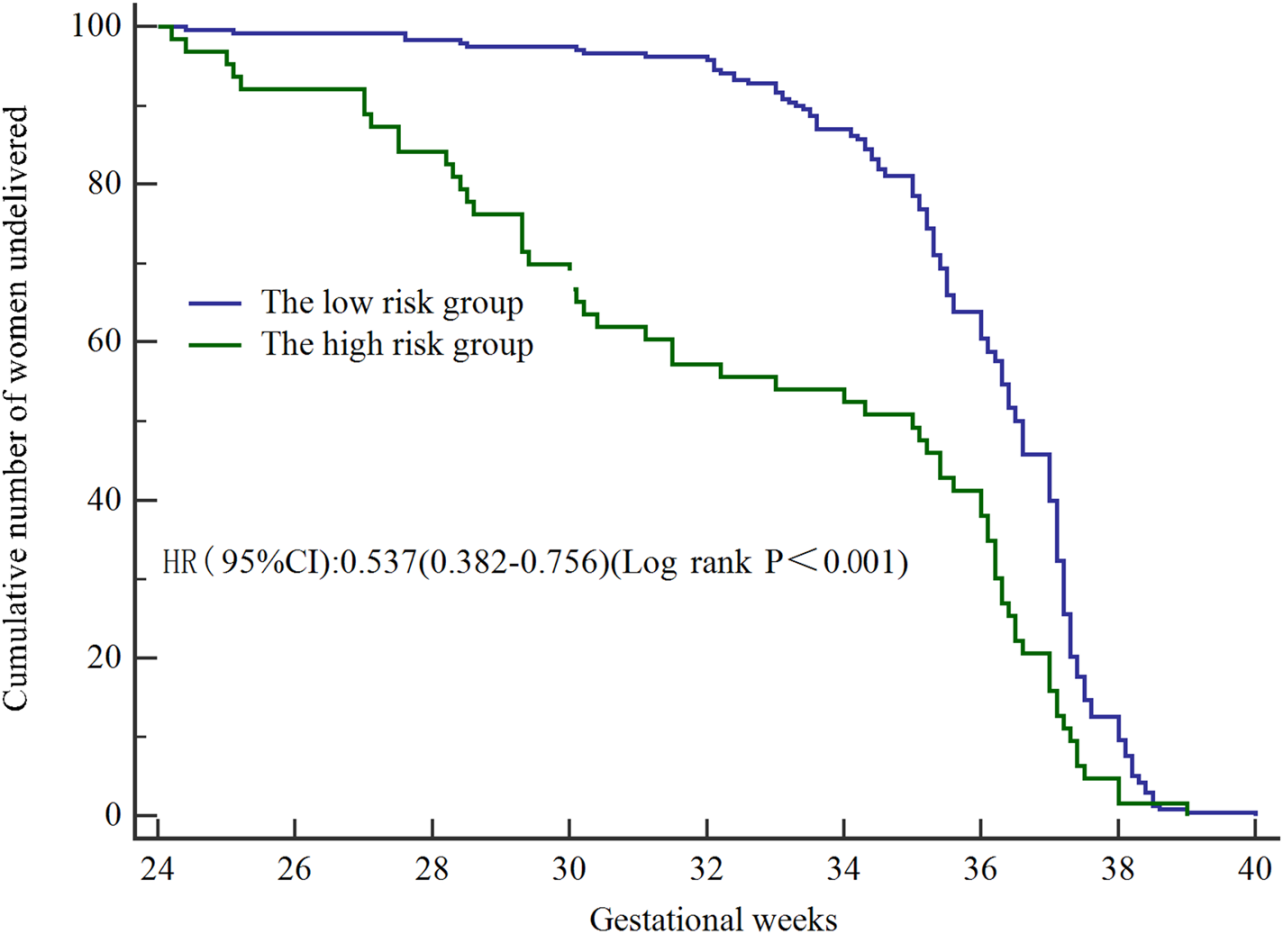
Survival curves of the high-risk group and the low-risk group in twin pregnancies. Kaplan–Meier curves were generated for GA at delivery. Log-rank test comparisons between the high-risk group and the low-risk group showed significant differences (HR 0.537, 95% CI 0.382–0.756, P < 0.001).

Next, the restricted cubic spline curve showed that the risk escalated continuously with the increasing scores obtained from the nomogram, which proves the reliability of the model (Fig. 6). In the training group and external validation group, the AUCs of the nomogram predicting the probability of SPTB at < 32 weeks were 0.848 (95% CI 0.809–0.892) and 0.782 (95% CI 0.735–0.826), respectively. In both groups, the prediction accuracy of the nomogram was superior to that of any single predictor (all P < 0.005) (Fig. 7). With the ROC curve of the training group, the optimal cut-off value of the risk score (125.16) was calculated based on the maximum Youden index. Then, the cut-off value categorized the training population into the low-risk group (155 twin pregnancies with risk score ≤ 125.16) and the high-risk group (572 twin pregnancies with risk score > 125.16) (OR 17.09, 95% CI 10.28–28.62, P < 0.05). The model reached a sensitivity of 80.00%, specificity of 88.17%, positive predictive value (PPV) of 50.33% and negative predictive value (NPV) of 96.71%. By using the same cut-off value in the external validation group, the results also proved the predictive performance of the nomogram (Table 3). Thus, we observed that the probability of SPTB in the high-risk group was significantly higher than that in the low-risk group (HR 0.537, 95% CI (0.382–0.756), P < 0.001), and gestational age at delivery was significantly earlier in the high-risk group (Fig. 8).

**Table 3:** Association between total risk scores and risks of PTB at < 32 weeks.

Models	Cut-off score	AUC	Groups	OR (95%CI)	TP	FP	FN	TN	Sensitivity (%)	Specificity (%)	FPR (%)	FNR (%)	PPV (%)	NPV (%)
Training group	125.16	0.848	Low risk	1.00	76	75	19	559	80.00*	88.17*	11.83*	20.00*	50.33*	96.71*
			High risk	17.09 (10.28–28.62)^a^										
External validation group		0.782	Low risk	1.00	27	35	8	216	77.14*	86.06*	13.94*	22.86*	43.55*	96.43*
			High risk	16.26 (9.61–27.38)^a^										

*TP* true positive, *FP* false positive, *FN* false negative, *TN* true negative, *FPR* false positive rate (= 1-specificity), *FNR* false negative rate (= 1-sensitivity), *PPV* positive predictive value, *NPV* negative predictive value.

^a^Adjustment for age, smoking, assisted reproductive technology, history of previous cervical surgery, gestational diabetes.

*p-value < 0.05.

## Discussion

In our retrospective analysis and external validation study, we developed a predictive model of SPTB at < 32 weeks based on maternal characteristics and sonographic cervical measurements to provide an accurate and comprehensive risk estimation, which can serve as an assessment tool to help physicians make decisions about further management of twin pregnancy.

The reason we comprehensively considered all the above factors when building the model was that the predictive performance of a single maternal factor or cervix geometry (including length) is not satisfactory, primarily due to poor sensitivity^33–37^. The mechanism of SPTB involves various mechanical stimuli (two continuously growing foetuses and the expanding uterus) and biochemical stimuli (inflammatory factors, fetoplacental signals and steroid hormones)^38,39^. Compared to that in singleton pregnancies, the mechanism of SPTB in twin pregnancies is predominantly determined by overdistension, whereas the role of inflammation and microbiologic invasion of the amniotic cavity (MIAC) is relatively minor^16^. Overdistension of the lower uterine segment and smooth muscle stretch in the human cervix provokes proinflammatory cytokine secretion, and research on changes in the cervical microstructure has been published by Vink et al.^17,40,41^. Jose Villar et al. proposed the use of a phenotypic classification system of PTB that does not force any PTB into a predefined phenotype but instead relies on a new conceptual framework in which a maternal clinical phenotype of PTB potentially related to a certain perinatal outcome is characterized by all relevant conditions observed during pregnancy^18^.

A series of common clinical characteristics, such as age, race, BMI, history of PTB, previous uterine surgeries, and tobacco usage, may indicate the initial states and variations in the structure and function of the cervix, which contributes to the risk of cervical insufficiency^19,20,25–27,42,43^. All these risk factors have interconnected effects and a computational framework for changing and remodelling the cervix. Our study is concordant with existing research indicating that nulliparity, lower prepregnancy BMI, history of PTB or late abortion, chorionicity, cervical funnelling and shorter cervical canal increase the possibility of SPTB in twin pregnancies. However, there is no risk calculation yet for SPTB before 32 weeks, which still represents a population with a tenfold increased risk for perinatal mortality compared to twins at term^44^. Our research incorporated maternal characteristics and biophysical tests of both cervical length and funnelling to develop a dynamic nomogram model that reached favourable PPV and NPV. Given that women with twin pregnancies are at high risk of preterm birth, better PPV and NPV indicate a higher rate of clinical diagnosis accuracy and a lower incidence of misdiagnosis. Therefore, our model may better guide clinical strategies, such as therapy decision-making and follow-up schedules, and could reduce complications for clinicians related to excessive monitoring and administration resulting from an undefined or inherently subjective risk assessment. Thus, the ability to generate a risk assessment and present it in the form of a percentage for each patient will enable caregivers to schedule more frequent follow-ups or administer targeted interventions, such as antenatal corticosteroids and tocolytic therapy, as well as transfer to a tertiary medical centre for patients at higher risk while reducing overtreatment and unnecessary hospitalization for those at lower risk. On the other hand, in the study design for the negative trials regarding PTB intervention, only a few researchers screened out and followed high-risk twin pregnancies, which may introduce confusion regarding indications for the interventions and result in bias when comparing outcomes^7,10,11,45^. To some extent, a lack of good care during surveillance frequently makes the difference in RCTs. It would be interesting in the future to determine whether the use of this tool to assess the indications for interventions and stratify patients according to risk could improve outcomes.

Our study has some limitations. Most importantly, it is limited by its retrospective design. There is a possibility of confounding bias: patients with unmeasured or unobservable factors who were excluded may represent patients at higher risk, so our study might ignore the most clinically interesting population. Second, the study population in the two centres is limited to our own population (Asian), which limits generalizability to people of different races. For example, in many high-resource countries, the risk of PTB is associated with obesity and is not underweight^25,46^. However, this potential limitation may also be considered a strength. All women included in the study were followed up and treated only in the two tertiary medical centres, which limits the confounding factors associated with the heterogeneity in provider bias, such as clinicians’ experience, and differences in the process of monitoring and management for offering the intervention. Based on the model, researchers in other countries can make use of their own data on demographic characteristics to justify the odds for their population. The last limitation is that because of the incomplete data for cervical length before 20 weeks, our model may poorly predict very early PTB since we adopted cervical measurements during 20–24 weeks and applied the system relatively late for the high-risk population^47^. In the future, we should concentrate on earlier evaluation of our algorithm to prevent early mortality and severe morbidity.

In summary, we developed and validated a dynamic nomogram model to predict the individual probability of early preterm birth; this nomogram better represents the complex aetiology of twin pregnancies and hopefully improves our understanding of the indications for interventions and, therefore, our ability to predict when they will be needed.

## Materials and methods

### Study population

We retrospectively collected data from 1461 consecutively asymptomatic women with twin pregnancies in the Fujian Maternity and Child Health Hospital (with an annual delivery number of more than 20,000 and a specified preterm birth clinic for ambulatory patients) and the Fujian Provincial Hospital (with 2398 beds and an annual delivery number of more than 5000) from January 2017 to December 2019. This retrospective study was performed with approval from the Ethics Committee of the Fujian Maternity and Child Health Hospital and the Fujian Provincial Hospital (Ethical approval number: 2019-014). The data were anonymous, and the requirement for informed consent was therefore waived. The completion and reporting of the study was in accordance with STROBE guidelines.

Subjects with any of the following conditions were excluded: incomplete records, genetic or structural abnormalities of either foetus, stillbirth of one or two foetuses, gestational age at birth < 20 weeks, twin birth weight < 500 g, monoamniotic or monochorionic twin pregnancy complicated by twin transfusion syndrome (TTTS) or twin anaemia–polycythaemia sequence (TAPS), placement of cervical cerclage, use of vaginal progesterone, maternal or foetal indications for iatrogenic PTB at < 32 weeks, or delivery at a medical centre other than ours. Women who gave birth before 20 weeks were excluded because in most cases, these women were likely to represent a unique subgroup of women whose cervical changes would have been detected very early and would be extremely obvious. Additionally, these women would not have had their cervical measurement at the indicated gestational stage in our study period, which was a major part of our research. As a result, we excluded 448 patients who met the exclusion criteria, and thus, 1013 patients met the inclusion criteria.

We assigned 727 samples collected from the Fujian Maternity and Child Health Hospital as the training group and 286 samples collected from the Fujian Provincial Hospital as the external validation group. All samples were reassessed by two obstetricians according to the inclusion and exclusion criteria (the flowchart showing the derivation of the development cohort and validation cohort is presented in Fig. 1).

### Data collection

Medical records were surveyed retrospectively, and the following data were extracted from patients’ charts. Demographic characteristics included maternal age, prepregnancy body mass index (prepregnancy BMI), nulliparity, history of previous cervical surgery, history of tobacco usage, clinical data including validation of gestational age by first trimester ultrasound, chorionicity, history of previous preterm or late abortion (during 12–28 weeks), complications during pregnancy, use of assisted reproductive technology, cervical length (20–24 weeks) and cervical funnelling, and gestational age at delivery.

Gestational age was calculated from the last menstrual period (LMP) and confirmed by the foetal crown-rump length measurement at the first trimester ultrasonic scan. If a discrepancy of more than 7 days was observed, the sonographic gestational age was followed. Chorionicity was confirmed by identifying lambda and T signs with ultrasound imaging between 11^+0^ and 13^+6^ weeks of gestation^48^.

**Figure 9:**
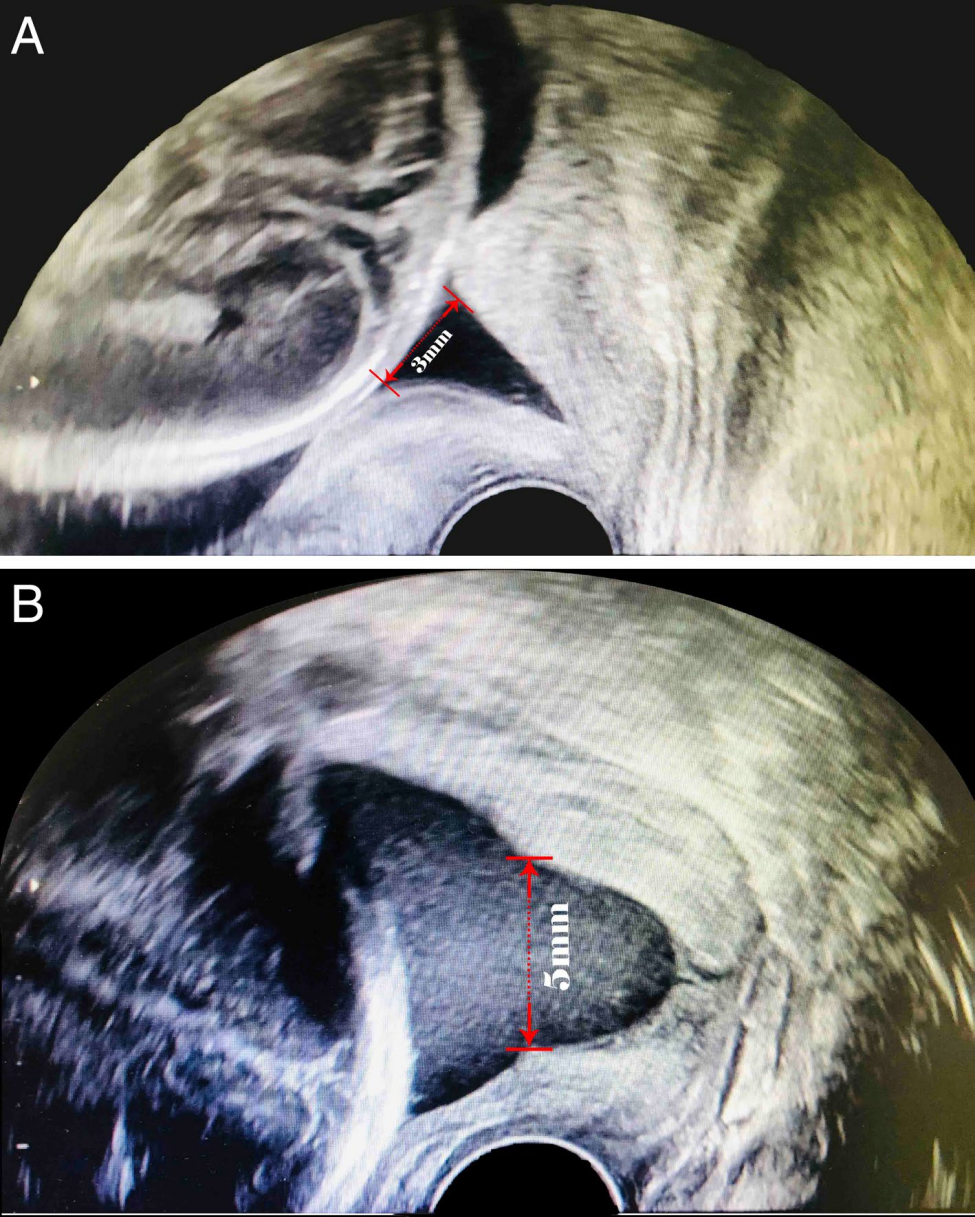
Cervical funneling detected by transvaginal ultrasound examination. (**A**) V-shaped funnel. (**B**) U-shaped funnel.

The ultrasound measurements were in accordance with a unified standard. All patients underwent transvaginal cervical length (TVCL) measurements between 20 and 24 weeks when the optimal image of the cervix was relatively easy to capture. The TVCL measurements of all subjects were performed by experienced sonographers at our ultrasound units. The ultrasound assessment was performed to measure the length of the cervical canal from the internal OS to the external OS and observe whether cervical funnelling appears with patients in the lithotomy position with an empty bladder. The measurement was repeated under gentle fundal pressure or the Valsalva maneuver unless severe cervical shortening was observed. Each examination was performed for at least 3 min as an evaluation period to detect the development of a “funnel”, which was defined as the protrusion of the amniotic membrane of 3 mm or more into the internal os as measured along the lateral border of the funnel (Fig. 9)^49,50^.

### Statistical analysis

#### Model development

Quantitative data are expressed as the median (interquartile range, IQR), and qualitative data are expressed as the number (percentage). The Wilcoxon–Mann–Whitney test or Fisher’s exact test was performed to measure the distribution differences of variables between the development and external validation groups. Univariate and multivariate logistic regression analyses were used to detect the correlation between clinical variables and preterm birth at 28 weeks, 32 weeks, and 34 weeks by applying a backward procedure based on the Akaike information criterion (AIC). By drawing the ROC curve of the predicted probabilities of SPTB before three gestational weeks (28, 32, 34 weeks) with multivariate meaningful variables, the prediction power for SPTB before the three gestational weeks was compared. Based on these results, a nomogram model with higher predictive performance was established, and bootstrapping techniques were used for internal verification to improve the robustness of the model.

#### Model validation

The performance of the nomogram models in identification and calibration was evaluated. The discriminative ability and predictive ability of the model were evaluated through Harrell's C-index, and external crowds were introduced to further evaluate the predictive value of the model. The calibration curve was analysed by drawing the predicted probability of the nomogram and the actual occurrence of SPTB. Restricted cubic splines were used to evaluate the correlation between the model's predicted score and the risk of SPTB. Kaplan–Meier curves were generated to compare the pregnancy outcomes in the two groups with different risk stratifications. ROC curve analysis was used to evaluate the prediction performance of the nomogram model and that of each meaningful parameter.

Statistical analyses were all performed with R 3.6.0 software (R: A Language and Environment for Statistical Computing, {R Core Team}, R Foundation for Statistical Computing, Vienna, Austria, 2018, https://www.R-project.org). A two-sided P-value < 0.05 was considered to indicate statistical significance.

### Ethics approval

All procedures performed in studies involving human participants were approved by the China Ethics Committee and the institutional ethical review boards of the Fujian Maternity and Child Health Hospital and the Fujian Provincial Hospital (Ethical approval number: 2019–014). Because the dataset contained no data enabling patient identification and all women received standard care, the study was exempt from informed consent requirements.
